# A Novel Polymer Film to Develop Heart Valve Prostheses

**DOI:** 10.3390/polym16233373

**Published:** 2024-11-29

**Authors:** Irina Yu. Zhuravleva, Anna A. Dokuchaeva, Andrey A. Vaver, Ludmila V. Kreiker, Alexandra B. Mochalova, Elena V. Chepeleva, Maria A. Surovtseva, Aleksei N. Kolodin, Elena V. Kuznetsova, Rostislav I. Grek

**Affiliations:** 1 E. Meshalkin National Medical Research Center of the RF Ministry of Health, 15 Rechkunovskaya St., Novosibirsk 630055, Russia; a_dokuchaeva@meshalkin.ru (A.A.D.); vaver_a@meshalkin.ru (A.A.V.); mochalova_a@meshalkin.ru (A.B.M.); e_chepeleva@meshalkin.ru (E.V.C.); mfelde13@yandex.ru (M.A.S.); ev_kuznetsova@meshalkin.ru (E.V.K.); 2 Research Institute of Clinical and Experimental Lymphology, Branch of the Federal Research Center Institute of Cytology and Genetics SB RAS, 2 Timakova St., Novosibirsk 630060, Russia; 3 Nikolaev Institute of Inorganic Chemistry, Siberian Branch of the Russian Academy of Sciences, 3, Acad. Lavrentiev Avenue, Novosibirsk 630090, Russia; kolodin.a.n@mail.ru; 4 Icon Lab Gmbh Ltd., 1 Barrikad St., Nizhny Novgorod 603003, Russia; grek@iconlab.ru

**Keywords:** biomaterials, heart valve engineering, hemocompatibility, biocompatibility, polymeric heart valves, REPEREN^®^ film

## Abstract

Polymer heart valves are a promising alternative to bioprostheses, the use of which is limited by the risks of calcific deterioration of devitalized preserved animal tissues. This is especially relevant in connection with the increasingly widespread use of transcatheter valves. Advances in modern organic chemistry provide a wide range of polymers that can replace biological material in the production of valve prostheses. In this work, the main properties of REPEREN^®^ polymer film, synthesized from methacrylic oligomers reinforced with ultra-thin (50 µm) polyamide fibers, are studied. The film structure was studied using scanning electron microscopy (SEM) and atomic force microscopy (AFM). The hydrophilicity and cytocompatibility with EA.hy926 endothelial cells were assessed, and a hemocompatibility evaluation was carried out by studying the platelet aggregation and adhesion upon contact of the REPEREN^®^ with blood. The mechanical behavior and biocompatibility (subcutaneous implantation in rats for up to 90 days, followed by a histological examination) were studied in comparison with a bovine pericardium (BP) cross-linked with an ethylene glycol diglycidyl ether (DE). The results showed that REPEREN^®^ films have two surfaces with a different relief, smooth and rough. The rough surface is more hydrophilic, hemo- and cytocompatible. Compared with the DE-BP, REPEREN^®^ has a higher ultimate tensile stress and better biocompatibility when implanted subcutaneously in rats. The key properties of REPEREN^®^ showed its potential for the development of a polymeric heart valve. Further studies should be devoted to assessing the durability of REPEREN^®^ valves and evaluating their function during orthotopic implantation in large animals.

## 1. Introduction

Surgeons all over the world widely use bioprosthetic heart valves, the leaflets of which are made of animal glutaraldehyde (GA)-fixed animal tissue (porcine aortic valve or bovine pericardium). Currently, the transcatheter implantation of aortic (TAVI) and mitral (TMVI) valves is becoming increasingly popular [1]. A transcatheter valve requires thinner leaflet material to pack the prosthesis into a delivery system of the smallest possible diameter. In this regard, the properties of porcine pericardium have been intensively studied in recent years [2,3,4,5,6]. However, all these xenogeneic materials have a common drawback: they have a high calcium-binding capacity in patients, which sooner or later leads to calcific dysfunction of the valve, especially in young patients [7,8,9]. Numerous approaches to supplementing the technology of GA preservation with various chemical and physical anti-calcification methods only slightly reduce the severity of the problem, without solving it radically [10,11].

More than 20 years ago, researchers began replacing the main cross-linking agent, GA, with epoxy compounds, ethylene glycol diglycidyl ether (DE) in particular. Since 1995, bioprostheses fixed with DE have been used in Russia. Experimental and clinical studies have shown that they are significantly more resistant to calcification than GA-treated material, but they also do not provide a 100% effect in patients [12,13,14,15].

The undoubted advantage of bioprostheses is that the design, with three highly mobile leaflets, provides good hemodynamics. The satisfactory hemocompatibility of xenogeneic materials makes it possible to abandon lifelong anticoagulation [16].

In this regard, the idea of replacing animal valve tissue with a thin, non-degradable polymer film that is resistant to calcification and cyclic loads, has good bio- and hemocompatibility, and mechanical properties close to natural materials seems very attractive. This idea has existed since the 1950s and led to the development of the “first generation” of polymer heart valves made of polysiloxanes, polyurethanes, and expanded PTFE (ePTFE). However, the materials available at that time did not have sufficient tensile strength and resistance to cyclic loads, degraded in the body due to oxidation and enzymatic hydrolysis, and could not reduce the risk of thromboembolic complications and calcification [17,18]. ePTFE was the most popular among cardiac surgeons, but the clinical results obtained did not meet expectations, even in such a low-load position as the pulmonary artery valve. High rates of dysfunction have been associated with stenosis, prosthetic endocarditis, and calcification [19,20].

Currently, many authors agree that the most promising modern materials for developing heart valve leaflets are as follows:

(1) Polyhedral oligomeric silsesquioxane poly(carbonate–urea) urethane (POSS-PCU);
(2) Siloxane poly(urethane–urea) (SiPUU, LifePolymer);
(3) Poly(styrene-b-isobutylene-b-styrene) (SIBS) and poly(styrene-b-4-vinylbenzocyclobuteneb-isobutylene-b-styrene-b-4-vinylbenzocylcobutene) (xSIBS);
(4) A nanocomposite graphene–PCU polymer (FGO-PCU, Hastalex) [17,18].

The POSS-PCU studies by A. Seifalian et al. led to the development of the traditionally designed tricuspid valves for surgical implantation (SAVR) and then the original transcatheter TRISKELE valve and its improved version, TRISKELION [21,22,23,24,25]. A POSS-PCU-based valve for TAVI was also developed by Mitrassist Lifesciences Ltd. (Shanghai, China), and it was first implanted in humans a year ago [26].

Research by a group of Australian scientists [27,28,29] devoted to SiPUU resulted in the Tria valves for SAVR and TAVI being manufactured by Foldax company (Salt Lake City, UT, USA). The clinical stage of studies began in 2022. These valves have been implanted in more than 200 patients in India, but there are no official publications on the mid-term results as yet [30,31]. The SAVR valves Polynova (PolyNova Cardiovascular Inc., Stony Brook, NY, USA) and Innovia (Innovia LLC., Miami, FL, USA), based on xSIBS, as well as Hastalex (NanoRegMed Inc., London, UK), based on FGO-PCU, are under active development [32,33,34].

In addition to the above-mentioned polymers, others also being tested for heart valves include: nanocomposite polyvinyl alcohol (PVA) and bacterial cellulose (PVA-BC), linear low-density polyethylene (LLDPE) and hyaluronan-enhanced linear low-density polyethylene (HA-LLDPE), triblock polyurethane combining siloxane and carbonate segments (Carbosil), and poly(styrene-b-ethylene/butylene-b-styrene) (SEBS) [17,18,32].

Polymeric valves are fabricated using various techniques to avoid the use of surgical sutures. For example, the Foldax Tria valve is fabricated by casting a solution onto a radiovisible polyetherether ketone stent, while the Hastalex valve is entirely made of polymers. Electrospinning and its variations (melt electrospinning and focused rotary jet spinning), as well as high-precision 3D printing [17,18,32,35], are considered promising methods. These are undoubtedly interesting approaches that open a new era in the development of heart valve devices. However, we believe it is possible to use a routine technique, involving the suture fixation of valve elements, similar to that used for pericardial valves. Surely, this requires the correct choice of suture material to avoid rupture of the polymer leaflets and calcification along the suture line.

The intensification of polymeric-valve development is due to the increased clinical requirements for the efficiency and safety of cardiac devices (on the one hand), and the success of modern organic chemistry in the synthesis of new materials (on the other). We have no doubt that, in addition to the listed compounds, other candidates for the role of heart valve leaflets will appear. Work with each of these candidates begins with the study of the material’s key properties, which determine the possibility of using it as a material for a heart valve prosthesis. These properties include the structure, mechanical properties, and hemo- and cytocompatibility of the material, cellular reactions to the material in vivo, and the structural integrity when in contact with the internal environment of the body. This is followed by testing the functional characteristics and resistance to high-cycle loads; transcatheter valves must be assessed when packaged in a delivery system, and the final stage of preclinical testing is orthotopic implantation in large animals.

When developing a valve prosthesis, the following matters must be taken into account. All heart valves experience different mechanical stresses, and the characteristics of the flows through the valves are also different. The closed mitral valve is subject to the greatest load (normally 100–140 mm Hg, and in arterial hypertension it can reach 200 mm Hg or more) at a moderate flow velocity (up to 1.5 m/s). The maximum peak flow velocity is observed when passing through the aortic valve (up to 2.5 m/s) at a lesser load (80–100 mm Hg). The loads and flow velocities inherent to the “right-side” valves (tricuspid and pulmonary) are several times less. So, depending on the intracardiac position of the prosthesis, the importance of this or that material property varies. For example, the aortic and mitral valves must be very strong to withstand destruction under high loads. At the same time, the high flow rate through the aortic valve makes the very high thromboresistance of the material not so important. It is known that, in the aortic position, even mechanical prostheses demonstrate satisfactory thromboresistance. On the contrary, for right-side valves functioning under conditions with low loads and low velocities, high strength is less important, but high thromboresistance is mandatory. However, we consider it inappropriate to search for a material for one specific valve. Our goal is to find a leaflet material that is strong, flexible, biocompatible, etc., to such an extent that it can be used for all types of valves.

In this paper, we report the first results obtained from studying the key properties of the REPEREN^®^ film that are required for its use in heart valve leaflets. REPEREN^®^ is the bioinert and biostable polymer approved in Russia for implantation into the patient’s body, but not into blood flow.

## 2. Materials and Methods

### 2.1. Materials

REPEREN^®^, catalog number R4-DA-80-80 (REPEREN^®^)-4-80-80 (IconLab Gmbh, Nizhny Novgorod, Russia), is a spatially cross-linked polymer synthesized from methacrylic oligomers reinforced with ultra-thin (50 μm) polyamide fibers and made in a sandwich style (polyamide fibers inside and REPEREN^®^ on both sides). The film has one ultra-smooth side and the other side is conditionally rough. We chose the 160 μm thick films to make the valves. Samples 80 mm × 80 mm were sterilized with ethylene oxide in “cold” mode (37 °C).

For a comparative analysis of the mechanical properties, biocompatibility, and calcium-binding capacity of REPEREN^®^, DE-cross-linked bovine pericardium was used.

#### Bovine Pericardium Treatment

Fresh pericardium was collected from a local slaughterhouse and transported to the laboratory. There it was cleared of surrounding tissue and washed several times with a sterile 0.9% NaCl solution. Preservation was carried out at room temperature with a 5% DE solution (0.1 M phosphate buffer, pH 7.4). For long-term storage of the pericardium, an alcohol solution was used. This solution contained 1,2-octanediol, phenoxyethanol, sorbic acid (1% in total), and 20% ethanol [36].

### 2.2. Non-Implanted Sample Studies

#### 2.2.1. Water Contact Angle Measurement

Measurement of the wetting contact angles on the films was carried out using an OCA 15 PRO (DataPhysics Instruments GmbH, Filderstadt, Germany) equipped with a measuring video system and a USB camera, as well as a high-aperture measuring lens with an adjustable viewing angle. All measurements were performed under standard conditions in a thermostatic box (T = 298 ± 2 °K and *p* = 1 bar). The needle diameter was 0.51 mm. Distilled water was used as the test liquid. The droplet volume was ~3.0 μL. The values of the contact angles were measured in the sessile drop mode. The observed contact angle was calculated using two algorithms: Ellipse-Fitting, and the Young–Laplace algorithm. The final values of the contact angles were calculated as the average of 4–5 measurements.

#### 2.2.2. Mechanical Testing

Uniaxial tensile testing was carried out using an ESM 303 machine (Mark-10 Corporation, Copiague, NY, USA) with a 0–100 N tensile gauge. The number of test samples was 20 pieces for each material. To prepare each sample, a cutting die with a working area of 10 mm in width and 27 mm in length was used. The BP samples were cut so that the superficial collagen bundles were oriented parallel to the testing axis, since such samples had higher ultimate stress values [37]. The fiber directions were determined visually according to the method proposed by R. Gauvin et al. [38]. The average value of measurements at 3 points, performed using a digital thickness gauge (Mitutoyo Corp., Kanagawa, Japan), was taken as the thickness of the sample (h, mm). The constant extension rate was 10 mm/min. All samples were stretched until failure.

The ultimate tensile stress (σ, MPa) was calculated as follows,

σ = F/(h × w)(1)

where F is the peak load prior to failure (N), h is the mean sample thickness (mm), and w is the width (10 mm) of the sample.

The failure strain (ε, %) was calculated as

ε = ∆L/L × 100(2)

where ∆L is the maximal deformation (mm) and L is the initial sample length (mm) between the grips.

The stress-strain curves of each group were plotted and analyzed using MATLAB 7.13 (The MathWorks, Natick, MA, USA).

Stiffness was evaluated by the elastic modulus (E, MPa) (Equation (3)), which was calculated for each sample at the points with the coordinates “maximal strain under failure stress” (“failure modulus”).

(3) $$E=\frac{\sigma }{\varepsilon }\times 100$$

#### 2.2.3. Hemocompatibility Study

To characterize the hemocompatibility of the films, platelet adhesion and aggregation were assessed.

##### Platelet Aggregation Test

For this study, a customized circulation system was used, consisting of a multichannel peristaltic UNAP pump (Mashintertorg, Russia), intravenous infusion systems as lines, and 50 ml Falcon plastic tubes (separate line and tube for each sample) (Figure S1A,B). REPEREN^®^ film pieces (83 mm × 25 mm) were placed with a smooth (n = 5) or rough (n = 5) surface in the lumen, and 3 Falcon tubes were left without films as controls. Whole blood obtained from healthy volunteers was mixed with 3.2% sodium citrate in a ratio of 9:1, respectively, and the system was immediately filled with the mixture. A sample of donor blood was also collected in a separate test tube to obtain initial data.

The circulation system functioned for 60 min at a temperature of 37 °C. The blood circulation rate was 0.04 L/min. Blood was collected from each test tube at 3 control time points (3, 30, 60 min). All blood samples were centrifuged at 200 g for 7 min to obtain platelet-rich plasma (PRP) (Figure S1C), then 0.3 mL of PRP was collected in special cuvettes and incubated at 37 °C for 3 min. The measurements were carried out using a laser platelet aggregation analyzer, ALAT-2 “Biola” (Biola, Moscow, Russia). Spontaneous (without any inducer), and induced (1 μM and 5 μM ADP) aggregation characteristics (aggregation maximum and aggregation rate) were assessed using the Born method for each sample.

##### Platelet Adhesion Test

After the circulation was completed, the film samples were carefully washed in a 0.9% NaCl solution by immersing them three times in a glass. The samples were then placed in Petri dishes with the test side up and treated with a 2% glutaraldehyde solution for 20 min. After fixation, the films were washed in a 0.9% NaCl solution for 1 min 3 times and dried at room temperature under aseptic conditions. The samples were then examined by scanning electron microscopy (see Section 2.4) at magnifications of ×100 and ×500 in at least 5 fields of view. The platelet density (the number of adherent platelets per 1 μm^2^ surface area) and the total covered surface area were assessed. All measurements were performed automatically using the KYKY SEM operating program (KYKY-EM6900LV v.1.8.1.2).

#### 2.2.4. Atomic Force Microscopy (AFM)

The objective of this study was to qualitatively and quantitatively characterize the smooth and rough surfaces of the REPEREN film. The works were carried out using an AF NTEGRA II microscope equipped with Nova-Px image analysis software (v.3.5.0) and an HA_FM A silicone ultrasharp cantilever (Spectrum Instruments, Russia) at a scan rate 0.7 Hz. Each smooth (n = 5) or rough surface (n = 5) was analyzed in the semi-contact mode in 10 fields. The root-mean-square (S_q_), maximum peak height (S_p_), maximum valley depth (S_v_), maximum height of the surface (S_z_), and mean summit curvature (S_sc_) were measured on 100 μm × 100 μm fields; the density of summits of the surface (S_ds_) was measured on 50 μm × 50 μm fields. Visualization of the adhered platelets was performed in a field of 8 µm × 8 µm.

#### 2.2.5. Cytocompatibility Evaluation

##### Materials for Cell Cultivation

An EA.hy926 cell line was made available by Dr. C. J. Edgell of Carolina University, USA. Cells were cultivated in Dulbecco’s Modified Eagle Medium/Nutrient F-12 (DMEM/F12) (Gibco, Carlsbad, CA, USA) medium supplemented with 10% FBS (Hyclone, Logan, UT, USA), 2 mM L-glutamine (ICN, Costa Mesa, CA, USA) and 40 μg/ml gentamicin sulfate (Dalkhimpharm, Khabarovsk, Russia) in a humidity-controlled environment with 5 vol% CO_2_ at 37 °C. During the process of passaging, the cells were grown in culture flasks and dissociated with Tripsin-Versene (Biolot, Saint Petersburg, Russia).

##### Evaluation of Cell Adhesion, Proliferation, and Viability

The EA.hy926 cells (5 × 10^4^ cells per sample) were placed on the smooth (n = 5) or rough (n = 5) surfaces of the sterile REPEREN^®^ samples and put into the 24-well plate wells. Subsequently, the cells were cultivated for a period of five and ten days. The identification of viable and non-viable cells was performed using fluorescent dyes, specifically acridine orange (DIA M, Russia; 100 µg/mL) and propidium iodide (Medigen, Novosibirsk, Russia; 100 µg/mL), respectively. Then the samples were incubated for a period of 10 min at 37 °C. Thereafter, the surface of each sample was imaged using an Axio Observer microscope (Zeiss, Oberkochen, Germany). Quantification of the cells was conducted using a minimum of five microscopic images of each surface, with a minimum of 500 cells counted in each image.

### 2.3. Implanted-Sample Studies

#### 2.3.1. Subcutaneous Implantation in Rats

All the experimental procedures were performed in accordance with the EU Directive 2010/63/EU for animal experiments and approved by the Ethics Committee of the E. Meshalkin National Medical Research Center.

Thirty 4-week-old Wistar rats (male, 40–50 g) were implanted with 3 samples of DE-BP and 3 samples of REPEREN^®^. The samples of both materials were cut using a round cutting die (6 mm in diameter).

Anesthesia was performed with 50 mg/kg Telazol (Zoetis Manufacturing & Research Spain, S.L, Gerona, Spain) via intramuscular injection. The surgical field was treated with a 10% povidone-iodine solution (Matyas kiraly ut 65, Kormend, Hungary) and 95% alcohol (Kemerovo Pharmaceutical Factory, Kemerovo, Russia). Three 3 mm skin incisions were made on the right side of the spine and three on the left. Then from these incisions, by pushing apart the surrounding tissue, subcutaneous pouches were formed. Each pouch was filled with one REPEREN or DE-BP sample and closed with one suture using Optilene 5/0 (BBraun, Rubi, Spain). Thirty samples of each biomaterial were explanted on days 30, 60, and 90. All the samples were rinsed with 0.9% NaCl (Solopharm, St. Petersburg, Russia).

Half of the samples for each material were prepared for histological studies and the other half underwent SEM and EDS examination.

#### 2.3.2. Histological Studies

All the samples prepared for histological studies were explanted with surrounding tissue capsules. The samples were fixed in 10% neutral buffered formalin for 24 h, paraffinized in an automatic histoprocessor (MT Point, St. Petersburg, Russia), and then embedded in paraffin for slide preparation (reagents and glass slides: Biovitrum, St. Petersburg, Russia). Slices, 6 μm thick, were prepared on a rotary microtome (Microm, Waldorf, Germany). All the samples were stained with hematoxylin and eosin (Biovitrum, St. Petersburg, Russia) following a standard protocol; for specific staining, a Russell–Movat pentachrome staining kit (Diapath, Martinengo, Italy) and a von Kossa staining kit (Biovitrum, St. Petersburg, Russia) were used, according to the manufacturer’s instructions. Light microscopy was carried out at ×50, ×100 and ×400 magnifications using an Olympus CX31 (Olympus, Tokyo, Japan) laboratory microscope with LCmicro 2.2 image analysis software (Olympus Soft Imaging Solutions GmbH, Münster, Germany). 

### 2.4. Scanning Electron Microscopy (SEM) and Energy Dispersive Spectrometry (EDS) Analysis

All the non-implanted or explanted samples were straightened out to a flat state, then dried aseptically at room temperature and coated with a 25–30 nm carbon layer using a GVC-3000 Thermal Evaporation Carbon Plating Instrument (KYKY Technology Co., Ltd., Beijing, China).

SEM and EDS analysis of selected areas were carried out with a WIN SEM A6000LV scanning electron microscope (KYKY Technology Co., Ltd., Beijing, China) equipped with an EDX AzTec One system and AztecOne 6.0 SP2 software (Oxford Instruments, Abingdon, UK). Sample images were obtained using secondary electron (SE) and back-scattering electron (BSE) detectors at an electron voltage of 20 keV and an electron beam setting of 120 µA. Ten fields were examined at 100×, 250×, 450×, and 700× magnification for each sample. 

### 2.5. Statistical Analysis

Statistical analysis was performed using STATISTICA 10.0 software (StatSoft Inc., Tulsa, OK, USA). The Shapiro–Wilk test was applied to check the normality of distribution in each group. Since the distribution of quantitative characteristics in most groups was not normal, non-parametric statistics were used, and the data are reported as medians (Me) and interquartile ranges (25–75%) (IQRs). The Mann–Whitney (M-W) U-test was used to compare the two groups. The significance level was set to *p* < 0.05.

## 3. Results

### 3.1. REPEREN^®^ Film Structure and Hydrophilicity

**Figure 1:**
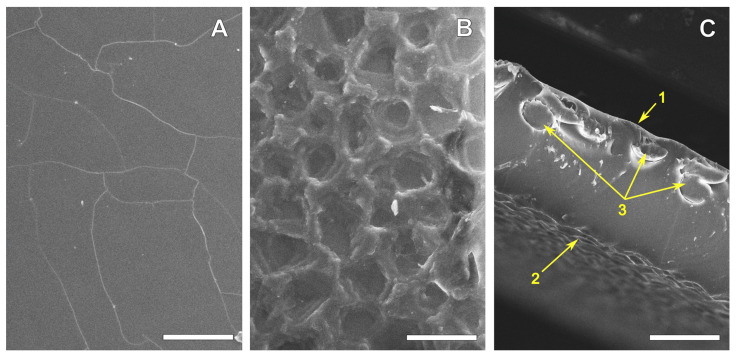
SEM images (SE) of the smooth (**A**) and rough (**B**) REPEREN^®^ film surfaces. Cross-sectional images (**C**): 1—smooth surface, 2—rough surface, 3—reinforcing fibers. Scale bars: 100 μm (**A**,**C**) and 50 μm (**B**).

The SEM images (Figure 1) clearly showed that one side of the film is smooth. The “cracks” on it appeared as a result of drying and carbon spraying during the sample preparation. The rough side had a “crater-like” relief with a “crater” depth of about 1.5–3 μm and a diameter of 15–50 μm. The reinforcing polyamide fibers in these 0.16 mm films were located closer to the smooth surface.

**Figure 2:**
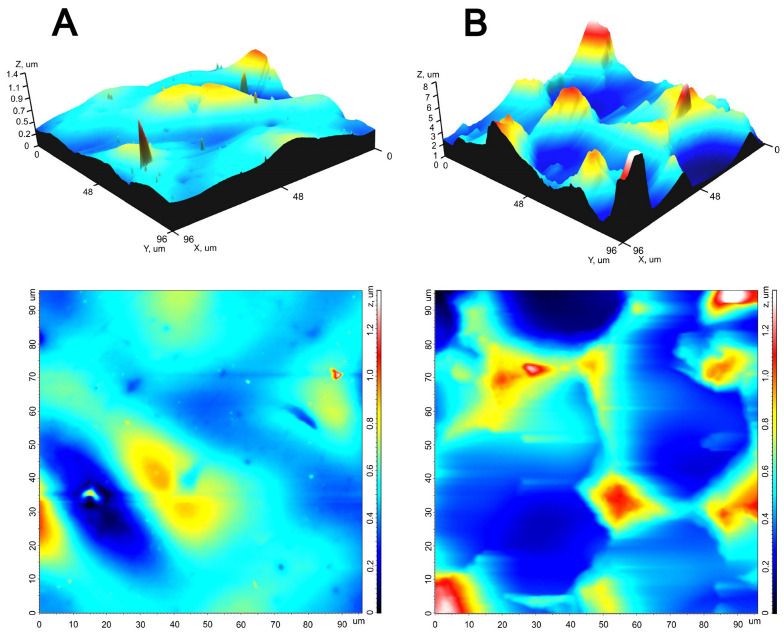
AFM images of the smooth (**A**) and rough (**B**) REPEREN^®^ film surfaces. The 3D (**top row**) and 2D (**bottom row**) colored images. Field sizes are 100 μm × 100 μm. Z-scales are 0–1.4 μm (**A**) and 0–8 μm (**B**).

The AFM results (Figure 2) allowed us to quantitatively characterize the relief of both surfaces (Table 1). The root-mean-square roughness, the height of peaks and the depth of valleys, and the maximum surface height of the rough surface exceeded similar values of the smooth surface by several times. The peaks on the smooth surface were sharp and mostly low, while those on the rough surface were smoothed out, forming a crater-like relief on this surface. The density of peaks (Sds) on both surfaces is extremely low, although on the smooth surface it is significantly higher, due to small sharp peaks.

The differences in the structures of the smooth and rough surfaces determined the differences in their hydrophilicity. Thus, a smooth surface is slightly hydrophilic, the contact angle of which can reach 80° or more (Table 2). The rough surface demonstrated the phenomenon of pseudohydrophobicity [39], due to air bubbles accumulating in the “craters” at the surface (see the image in Table 2), determining heterogeneous wetting. After the complete release of air bubbles and the transition to homogeneous wetting (Figure S2), the rough surface demonstrated a smaller water contact angle compared to the smooth surface.

### 3.2. Mechanical Features of the Materials

Under uniaxial tension, the REPEREN^®^ film demonstrated significantly higher ultimate tensile stress values (on average 3.5 times, *p* = 0.002) compared to BP (Table 3). The average values of the deformability of the samples are not statistically different (*p* = 0.12). The elastic modulus of the REPEREN^®^ at the point of rupture is 4.6 higher, which indicates its greater stiffness. We suppose that the high elastic modulus is largely due to the reinforcing fibers. Based on this, the problem of excessive stiffness can be solved by reducing the fiber diameter and/or weaving density.

**Figure 3:**
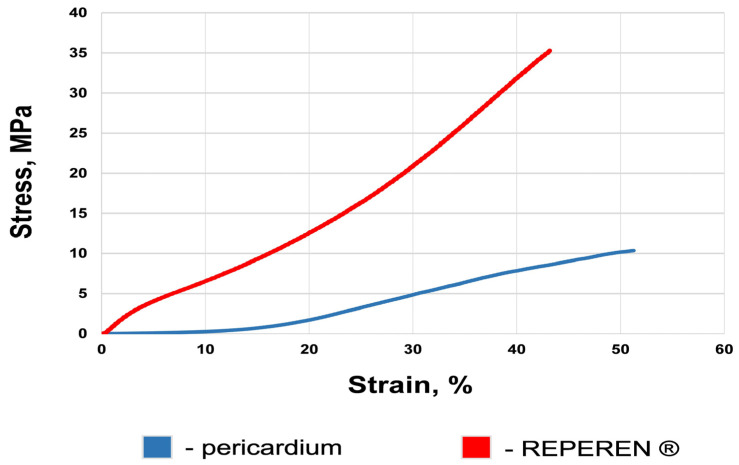
The typical stress/strain curves of the bovine pericardium and the REPEREN^®^ film.

The mechanical behavior of REPEREN^®^ is generally different from that of most polymers, where the stress in the material rapidly increases under uniaxial tension, followed by a plateau and the onset of plastic deformation [40,41]. The stress/strain curve of REPEREN^®^ resembles the behavior of biological materials with a phase similar to the elastic one (Figure 3). However, the typical curves of the film and the DE-pericardium are different (Figure S3). After the point of ultimate tension, a sharp drop in the film curves occurs (Figure S3A). The typical curves of the DE-pericardium feature the dentate increasing tension, in some samples, or a smooth decrease after reaching the point of ultimate load, in others (Figure S3B). These are due to the non-simultaneity of collagen rupture in the biological material.

### 3.3. Hemocompatibility

**Figure 4:**
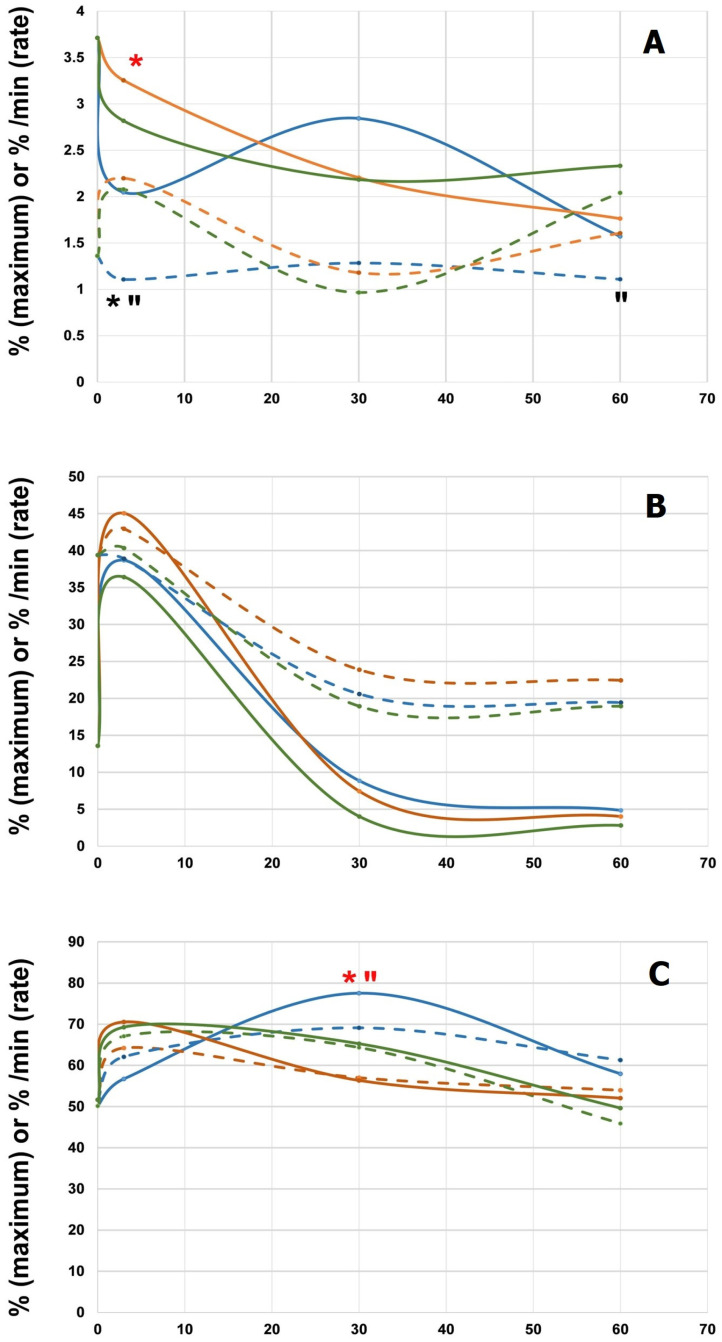
Platelet aggregation: spontaneous (**A**) and induced by ADP 1 μM (**B**) or 5 μM (**C**). Aggregation maximum (%, solid lines) and aggregation rate (%/min, dashed lines). The colors code the sample types in contact with the platelet-rich plasma: control (green), smooth (blue), and rough (brown) surfaces. Significant difference (*p* < 0.05) between control and tested samples (“) or between two experimental groups (*). Red icons mean differences in aggregation maximum, black ones mean differences in aggregation rate.

Figure 4 shows the dynamics of platelet aggregation during 60 min contact with blood. Of greatest interest were the results with spontaneous aggregation (Figure 4A). Thus, by the 3rd minute, we observed the lowest rate and the maximum on the smooth side of the film; then the rate of aggregation increased slightly with an increase in the maximum, but the maximum value did not differ significantly from the control and the rough-side samples. At the same time, the rate and maximum of aggregation on the rough side decreased by the 30th minute in the same way as in the control; the rate of aggregation increased in these groups by the 60th minute with an unchanged rate of aggregation on the smooth side. In this regard, after 60 min, the aggregation results of both film sides did not differ significantly from the control. These aggregation dynamics resulted in no significant differences between the groups by the 60th minute.

With induction by 1 mM ADP (Figure 4B) in all tested groups the curves of platelet aggregation were the same: an increase by the 3rd minute of contacting blood and a subsequent decrease. With induction by 5 mM ADP (Figure 4C) a significantly higher aggregation maximum was observed at the 30th minute on the smooth surface, again with the subsequent leveling of both indices in all three groups at the 60th minute.

**Figure 5:**
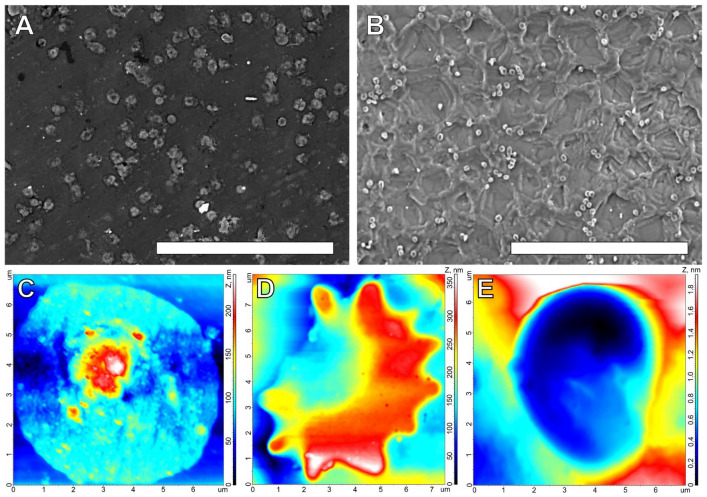
Platelet adhesion (BSE) on the smooth (**A**) and rough (**B**) surfaces. Scale bar 100 μm. AFM images of activated platelets (**C**,**D**) on the smooth surface and a non-activated platelet (**E**) on the rough surface.

Platelet adhesion on the smooth and rough surfaces differed, although the number of adhered platelets on the two different surfaces was comparable: 1026 (488; 1368)/mm^2^ on the smooth surface and 738 (356; 1000)/mm^2^ on the rough surface (*p* = 0.327). However, the SEM images (Figure 5A,B) clearly showed that the diameter of platelets on the smooth surface was 1.5–2 times larger. AFM visualization proved that the platelets adhered to the smooth surface were mainly activated, as they were spread (Figure 5C) or had filodopodia (Figure 5D). The platelets adhered to the rough surface had a normal disc shape, characteristic of non-activated platelets.

### 3.4. Cytocompatibility (In Vitro Test)

We found distinct differences between the smooth and rough surfaces when evaluating compatibility with the EA.hy926 endothelial cells.

**Figure 6:**
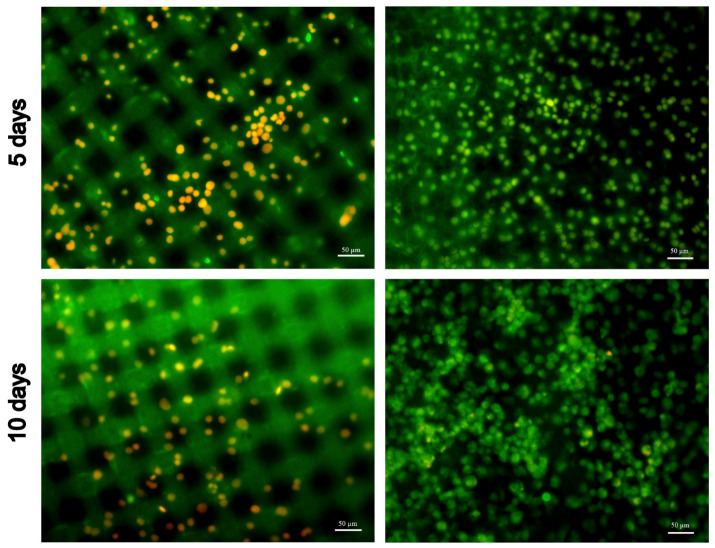
Fluorescence microscopy images of the EA.hy926 cells on the smooth (**left column**) and rough (**right column**) surfaces of the REPEREN^®^ samples after 5-day (**top row**) and 10-day (**bottom row**) culturing. Staining was performed with acridine orange (green, live cells) and propidium iodide (red, dead cells). Autofluorescent reinforcing polyamide fibers are visible through the smooth surface. Scale bar: 50 μm.

The number of adhered cells after 5 days was notably less on the smooth surfaces than on the rough ones (Figure 6). Moreover, most of the cells were dead or apoptotic. On the rough surface, by this time, a continuous layer of viable endothelium has already begun to form. The EA.hy926 cells did not proliferate on the smooth surface, so by the 10th day their number had not increased, or even decreased, whereas on the rough surface the cells actively proliferated and retained their viability.

### 3.5. Results of Subcutaneous Implantation

Above all, we did not detect calcification, either in the studied film or in the DE-treated BP, up to 90 days of implantation. However, the cellular reaction from the surrounding tissues was completely different.

**Figure 7:**
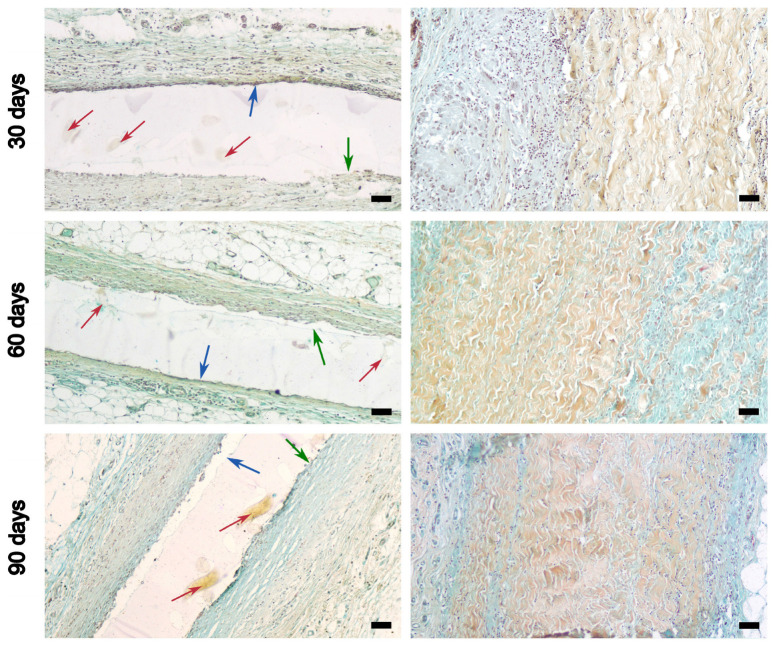
Tissue reaction to the REPEREN^®^ film (**left column**) and the DE-treated bovine pericardium (**right column**) implanted subcutaneously in rats. Movat pentachrom staining: The arrows point to the smooth (green) and rough (blue) surfaces and the reinforcing polyamide fibers (red). Scale bar: 50 μm.

Active cellular infiltration is a feature of xenogeneic pericardium at all time points, but the greatest number of lymphocytes and macrophages were detected on the 30th day. Already at this stage, cells penetrated from both the fibrous and serous surfaces between the bundles of collagen fibers, gradually separating them (Figure 7 and Figure S4). This visually “blurs” and increases the thickness of the peri-implant capsule. Since REPEREN^®^ is a nonporous film, the cellular reaction was bordered by its surfaces. A greater accumulation of cells was observed at the boundary with a rough surface, while cells were almost absent at the boundary with a smooth surface. In general, on the 30th day, the number of cells was small; a thin connective tissue capsule formed around the film, in which fibrillar elements predominated over cellular ones.

By day 60, the tissue around the implanted film demonstrated an even more meager cellular reaction, and the capsule began to vascularize and thin. In DE-BP, the cellular reaction also weakens, but the cells that have penetrated between the collagen bundles are transformed (apparently into fibroblasts) and begin to synthesize connective tissue de novo. This leads to an even greater loss in the structure of the collagen framework in the BP.

On the 90th day, the film was covered on both sides with a thin fibrous capsule with poor cell content. In the DE-BP implant, the cellularity of the capsule also decreased, but the volume of newly formed connective tissue deep in the implant increased, and the degradation of its own collagen matrix intensified.

**Figure 8:**
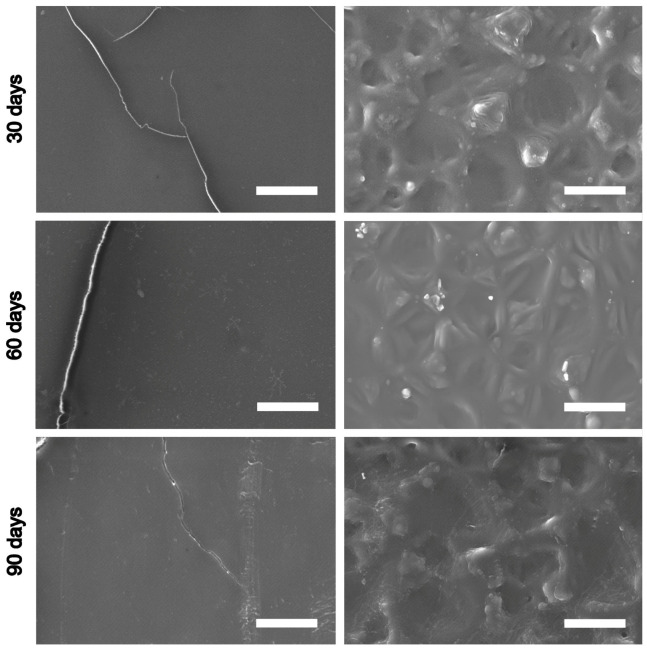
SEM images (SE) of the smooth (**left column**) and rough (**right column**) surfaces of the REPEREN^®^ film after subcutaneous implantation in rats. Scale bars are 30 μm.

When examined by SEM, the explanted REPEREN^®^ samples demonstrated an unaltered surface structure (Figure 8), regardless of the implantation time. After 30, 60, and 90 days, no defects or signs of calcification were found on the smooth surface. The rough surface retained a crater-like relief without any signs of degradation. The crystals occasionally detected on the surfaces (see 60 days, rough surface) are deposits of NaCl, which is used when washing the explants. This is confirmed by the EDS data (Figure S5).

## 4. Discussion

REPEREN^®^ is quite a new material. It was developed for reconstructive surgery on the dura mater and anterior abdominal wall and is also used for anti-adhesion patches in abdominal surgery. Previous clinical experience shows it to be a bioinert and biostable material. REPEREN^®^ medical products are reinforced or non-reinforced films of various thicknesses, that can be perforated or non-perforated patches. Some products have a special shape corresponding to the implantation zone [42]. All of them are united by the general name REPEREN^®^. Each product has a unique catalogue number, depending on individual characteristics. However, none of the REPEREN^®^ varieties have ever been used for cardiovascular surgery, much less for developing a polymer valve. Our study is the first in this direction. We chose reinforced R4-DA-80-80 (REPEREN^®^)-4-80-80 to study.

Considering that aortic and mitral prostheses must be very strong to resist destruction under high loads, the polymer leaflet must not be less resistant than traditional xenogeneic materials [17,18]. Thus, under uniaxial tension testing, its ultimate tensile stress should be at least no less than that of modern models of bioprostheses: 25–30 MPa. Such strength characteristics (from 30 to 57 MPa) are found in polymer films made of POSS-PCU (TRISKELE valve), SiPUU (Tria Foldax valve), xSIBS (Innovia valve), and FGO-PCU (Hastalex) [18,31]. However, these materials have an extremely high strain (400–1000% [18]), which seems excessive in our opinion. The strain of natural aortic cusps and bioprostheses made from bovine or porcine pericardium never exceeds 50–90% [4,37,38]. This is beneficial for normal valve function. When the highest hydraulic pressure is applied to a closed valve, the leaflets should damp the load but should not overstretch or inflate. The strain of REPEREN varies between 35% and 59%, but the median (40%) does not differ significantly from that of DE-BP (49%). We see ways to increase the strain by 20–30% by improving the reinforcing fibers. Besides, when selecting a material for transcatheter valves, it is necessary to keep in mind that the thinner the leaflet material, the easier it is to fold into a delivery system. The average thickness of cross-linked bovine pericardium is 0.3–0.6 mm, while for porcine it is 0.2–0.25 mm. REPEREN^®^ film, which was used in this work, is 0.16 mm. Among the polymers tested as valve leaflets, samples less than 100 μm are also known [17], but the sandwich structure of REPEREN^®^, with reinforcing fibers of 50 μm in diameter, somewhat limits the possibilities of reducing the film thickness.

Since the REPEREN^®^ film is manufactured by the casting method, it has two surfaces that differ in their properties: one smooth and one rough. The smooth surface is low-hydrophilic, with its water contact angle approximately corresponding to xSIBS or FGO-PCU. The rough side (after reaching the homogeneous wetting phase) is more hydrophilic and corresponds to SIBS [18].

Differences in the structure and hydrophilicity of the surfaces determine their differences in hemo- and cytocompatibility. The rough surface is more “friendly” to endothelial EA.hy926 cells, as evidenced by the active adhesion and proliferation, as well as the high viability of these cells (Figure 6). Previously, we reported that cell adhesion and proliferation are difficult on very smooth surfaces [43], while at a moderate height and density of smoothed peaks, cells engulf nanopillars with a medium adhesion depth without membrane penetration [44]. The existence of low but sharp peaks on the smooth surface (Figure 2) not only determines the poor survival of the EA.hy926 cells, but also promotes the activation of platelets, their spread and change in shape (Figure 5), caused by the activation of cellular signaling, followed by a cascade of cytoskeleton reorganization processes [45,46]. Meanwhile, the hemocompatibility of the valve material is of greatest importance at low blood flow velocities. Clinically, this is manifested as a higher incidence of thrombosis and thromboembolism in the right-side positions (tricuspid and pulmonary valves). Under slow blood flow, platelets have more opportunities for adhesion and activation on the surface of the prosthesis. This is why we chose a very low flow rate (below physiological) to assess the platelet aggregation and adhesion. We found that the numerical characteristics of platelet aggregation and adhesion were almost the same on both surfaces. At the same time, the detected activation of platelets on a smooth surface encourages us to further study the REPEREN^®^ hemocompatibility in more detail.

The REPEREN^®^ manufacturers rightly declare it as a bioinert material [42]. When comparing the tissue reaction to subcutaneous implants of REPEREN^®^ and DE-treated BP in rats, it was shown that the film is more biocompatible (we can even talk about bioinertness). On the 30th day, the inflammatory cellular reaction to it is relatively scanty, and by the 90th day it is almost absent; the connective tissue capsule is thin, but completely isolates the implant from the surrounding tissues. In contrast to the film, pronounced infiltration by lymphocytes and macrophages was observed in DE-BP on the 30th day, with the cells penetrating deep between the collagen bundles (Figure 7 and Figure S4). On the 90th day, this led to moderate sprouting of the border zones of the implant with newly formed connective tissue. When interpreting these results, it should be taken into account that DE-cross-linked xenogeneic material is less immunogenic and causes a smaller cellular response compared to its GA-treated counterpart [15,37].

The REPEREN^®^ film structure is stable for 90 days of subcutaneous implantation and does not appear to undergo hydrolysis or enzymatic lysis (Figure 8). However, the subcutaneous model may differ greatly from the circulating blood model in biodegradation promoters. Therefore, the final answer about the biodegradability or non-biodegradability of REPEREN can be given only after a long-term experiment on large animals to obtain infra-red spectra of the material before and after implantation for different periods.

## 5. Conclusions

This study reveals that the key properties of REPEREN make it a promising candidate for the development of a polymeric heart valve. The results showed that REPEREN^®^ films have two surfaces with different reliefs, smooth and rough. The rough surface is more hydrophilic, hemo- and cytocompatible. Compared with DE-BP, REPEREN^®^ has a higher ultimate tensile stress and better biocompatibility when implanted subcutaneously in rats. No signs of polymer biodegradation were observed for up to 90 days in the subcutaneous model. The next steps of our work will be an in vitro study of the functioning and durability of REPEREN valves, as well as the evaluation of the behavior of these valves after orthotopic implantation in large animals in various intracardiac positions.

## Figures and Tables

**Table 1:** Numerical characteristics of the surface roughness and peaks (field sizes are 100 μm × 100 μm).

Parameter	Smooth Surface	Rough Surface	*p*
Sq, μm	0.20 (0.15; 0.24)	1.40 (1.31; 1.63)	0.0001
Sp, μm	1.32 (1.21; 1.41)	5.92 (5.62; 7.56)	0.0026
Sv, μm	0.76 (0.65; 0.87)	2.78 (2.39; 3.30)	0.003
Sz, μm	2.08 (1.86; 2.28)	9.02 (8.01; 10.85)	0.00001
Sds, 1/μm^2^	0.04 (0.03; 0.07)	0.01 (0.01; 0.02)	0.0015
Ssc, 1/μm	0.15 (0.12; 0.15)	0.64 (0.41; 0.85)	0.001

**Table 2:** Water contact angle.

Surface	Contact Angle, °		Drop Image
	Ellipse-Fitting	The Young-Laplace Algorithm	
Smooth, 0 s	82 ± 3	82 ± 4	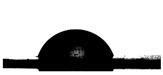
rough, 0–38 s	89 ± 6	91 ± 5	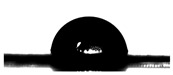
rough, 60 s	77.2 ± 0.3	76.3 ± 0.6	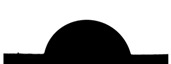

**Table 3:** The main mechanical characteristics * of DE-BP and the REPEREN^®^ film.

Parameter	DE-Treated BP	REPEREN^®^ Film
Ultimate tensile stress, MPa	10.3 (9.0; 12.5)	35.7 (31.4; 40.7)
Deformability, %	49.9 (44.7; 56.2)	40.0 (32.0; 59.7)
Elastic modulus	17.5 (14.6; 21.6)	81.8 (65.6; 95.8)

* the data are presented as Me (IQR).

## Data Availability

The data presented in this study are available on request from the corresponding author, due to the privacy agreement between the authors and the E. Meshalkin National Research Center.

## Supplementary Materials

The following supporting information can be downloaded at https://www.mdpi.com/article/10.3390/polym16233373/s1: Figure S1: The customized circulation system (A,B) and platelet-rich plasma ready to measuring platelet aggregation (C); Figure S2: The dynamics of the water contact angle (a) and the drop base diameter (b) on the rough surface: 0–38 s is a phase of heterogeneous wetting, 38–45 s is the transition period of air bubbles coming out, 45–80 is a phase of homogeneous wetting; Figure S3: The stress/strain curves of REPEREN (A) and DE-preserved bovine pericardium (B); Figure S4: DE-treated bovine pericardium implanted subcutaneously in rats, 30 days. H&E staining, scale bars 50 μm (A) and 20 μm (B). Green arrows point to degrading collagen bundles surrounded by lymphocytes; Figure S5: EDX spectrum of the rough surface elemental map (REPEREN sample from Figure 8; 60 days after implantation).
