# Supplementary material for: A Novel Polymer Film to Develop Heart Valve Prostheses
Source: Polymers (Basel). 2024 Nov 29;16(23):3373. doi: 10.3390/polym16233373 (PMC11644251; doi:10.3390/polym16233373)
Supplement: Supplementary file 1 [file polymers-16-03373-s001.zip › polymers-3312020-supplementary.pdf]

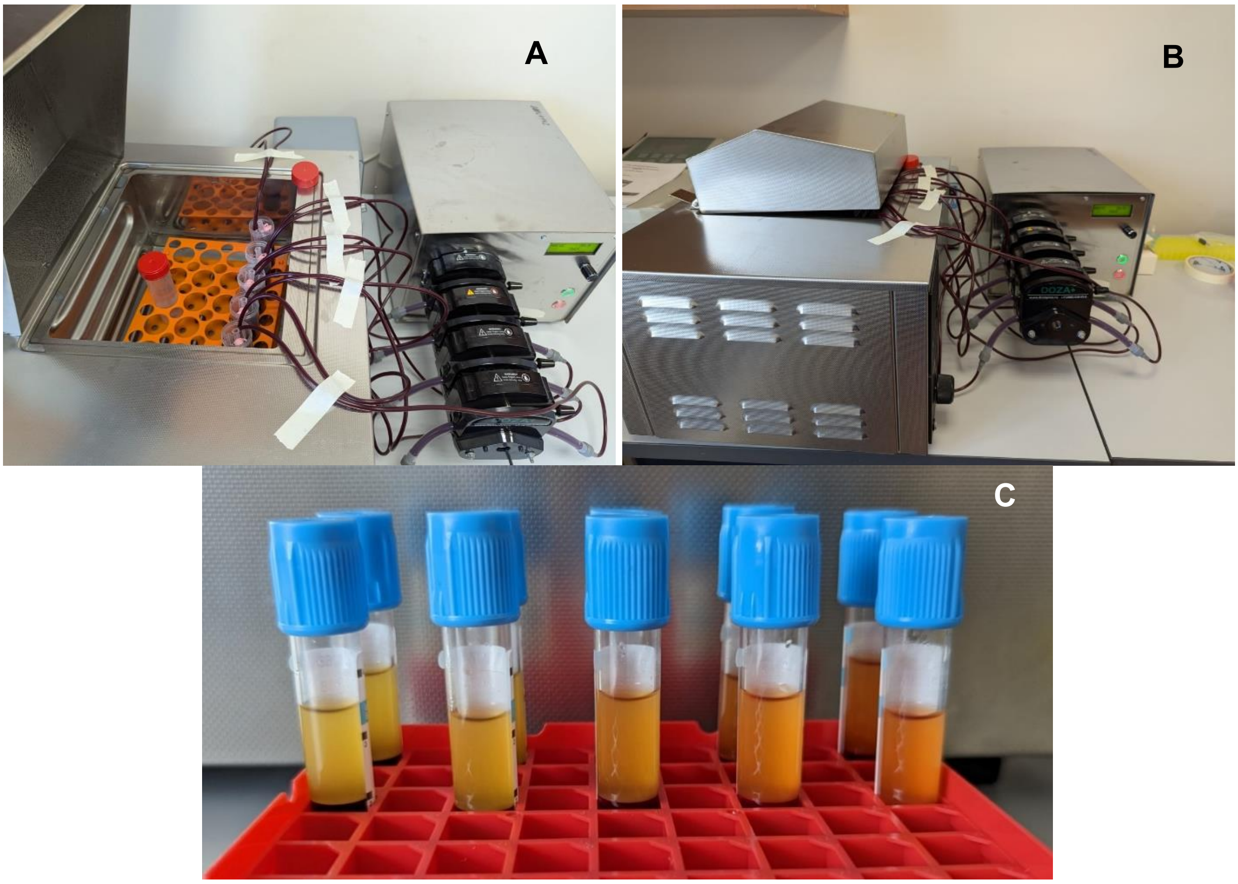

Figure S1. The customized circulation system (A and B) and platelet-rich plasma ready to measuring platelet aggregation (C).

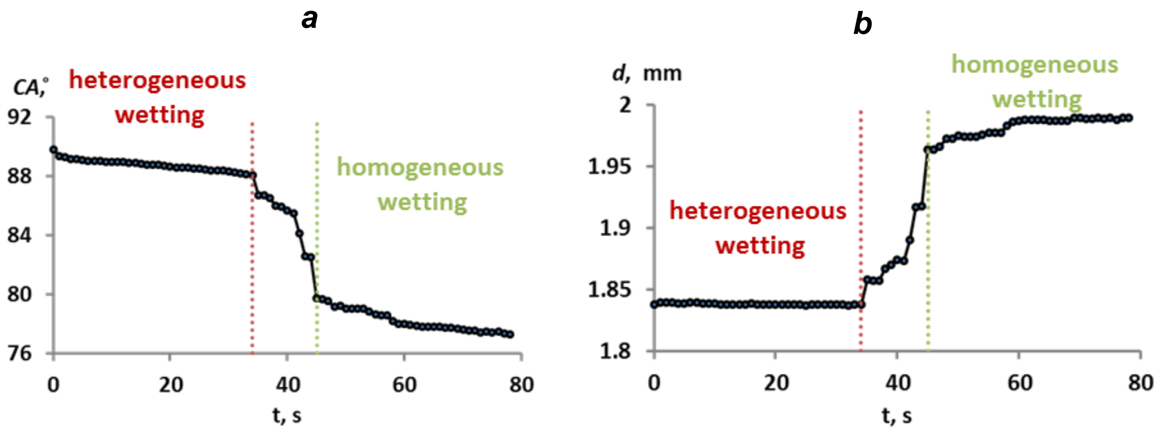

Figure S2. The dynamics of the water contact angle ***(a)*** and the drop base diameter ***(b)*** on the rough surface: 0-38 sec is a phase of heterogeneous wetting, 38-45 sec is the transition period of air bubbles coming out, 45-80 is a phase of homogeneous wetting.

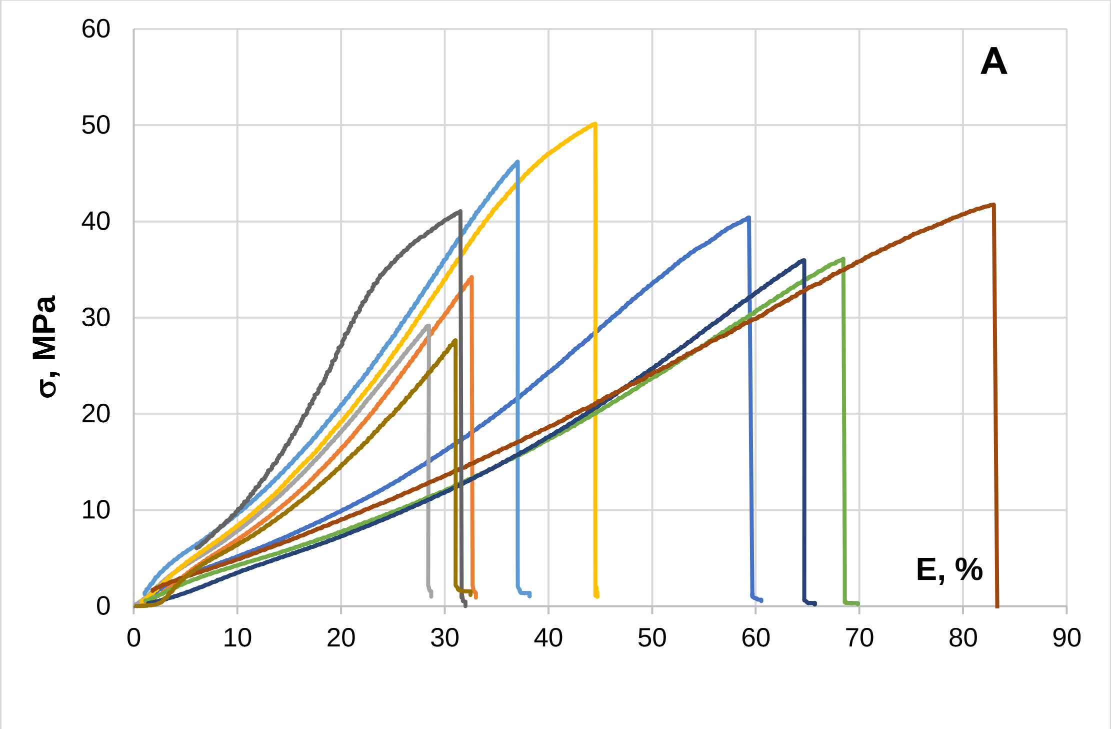

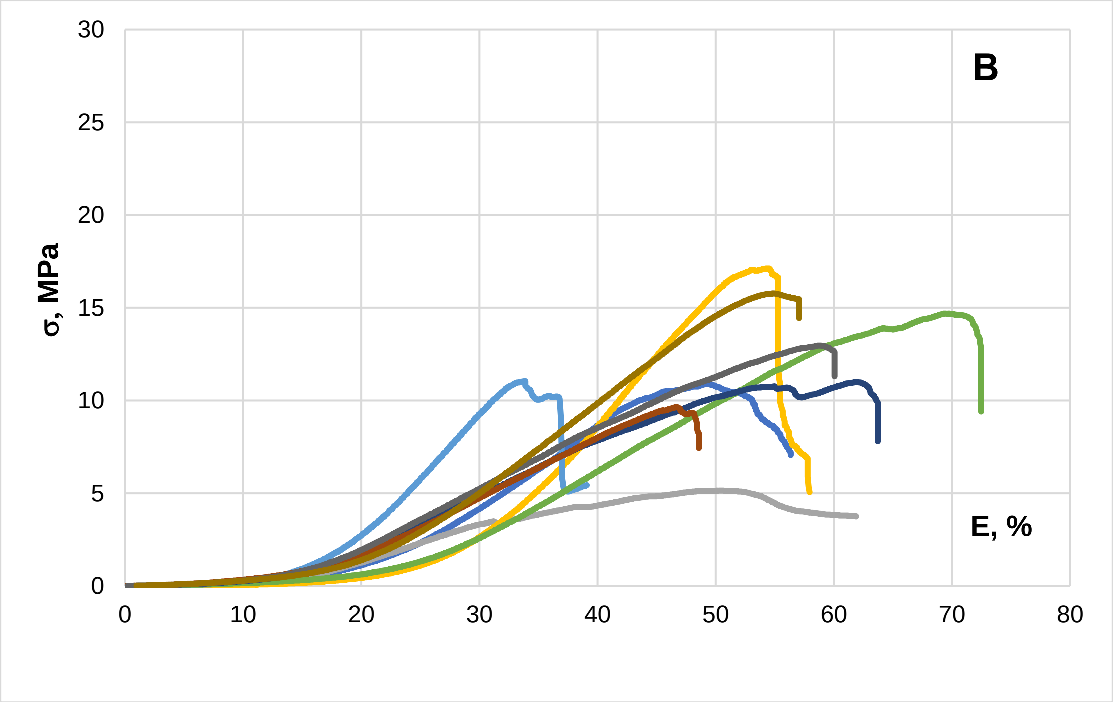

Figure S3. The stress/strain curves of REPEREN (A) and DE-preserved bovine pericardium (B)

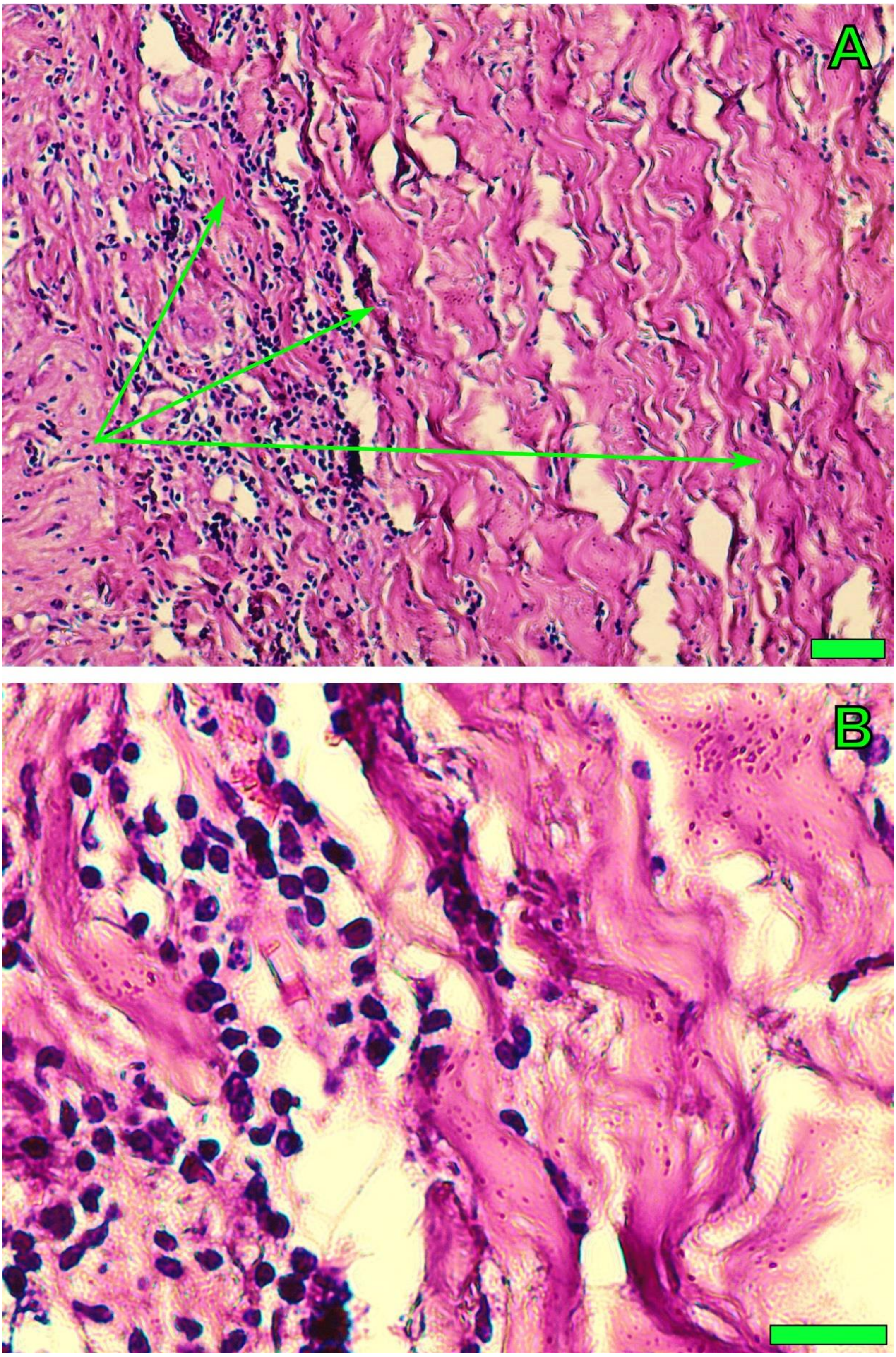

Figure S4. DE-treated bovine pericardium implanted subcutaneously in rats, 30 days. H&E staining, scale bars 50 µm (A) and 20 µm (B). Green arrows point to degrading collagen bundles surrounded by lymphocytes.

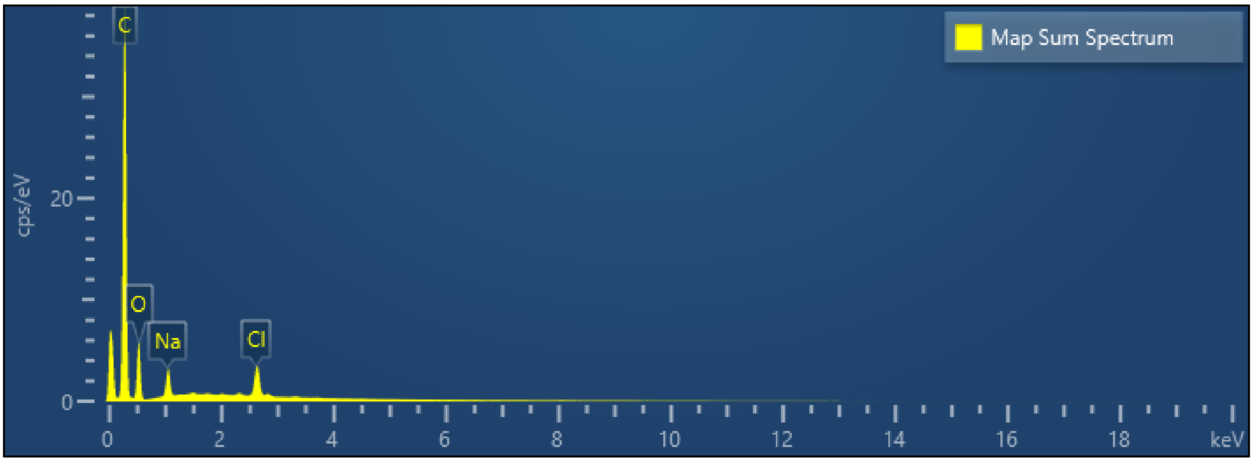

Map Sum Spectrum				
**Element**	**Line Type**	**Weight %**	**Weight % Sigma**	**Atomic %**
C	K series	77.81	0.07	83.14
O	K series	19.32	0.07	15.50
Na	K series	1.62	0.01	0.90
Cl	K series	1.25	0.01	0.45
Total		100.00		100.00

Figure S5. EDX spectrum of the rough surface elemental map (REPEREN sample from figure 8; 60 days after implantation).
